# Editorial: Language and Mild Cognitive Impairment

**DOI:** 10.3389/fpsyg.2020.02264

**Published:** 2020-10-07

**Authors:** Arturo X. Pereiro, Carlo Semenza, Onésimo Juncos-Rabadán

**Affiliations:** ^1^Department of Developmental Psychology, University of Santiago de Compostela, Santiago de Compostela, Spain; ^2^Department of Neuroscience, University of Padua, Padua, Italy

**Keywords:** mild cognitive impairment, lexical-semantic abilities, language, early detection, dementia

In recent decades there has been extensive research into the early detection of dementia, particularly, Alzheimer's disease (AD). Descriptions of the cognitive continuum from normal aging to dementia have identified two early states (Jack et al., 2018). The earliest, Subjective cognitive decline (SCD), is characterized by the presence of significant and persistent cognitive complaints (Jessen et al., 2014). The next state, Mild cognitive impairment (MCI), is a condition in which individuals show objective impairment in cognition with minimal impairment of their capacity to undertake the instrumental activities of daily living (Petersen et al., 2018). The main aim of research on SCD and MCI was to increase the validity of diagnostic criteria and identify behavioral, neuropsychological, and biological markers that allow for a reliable and valid prediction of the progression and risk of dementia (Jack et al., 2018).

Meta-analytical research (Belleville et al., 2017) has reported that measures of verbal memory and language, particularly from tasks requiring lexical production, such as fluency and naming, are among the main neuropsychological predictors of MCI and its progression to AD. With the aim of providing an overview of recent contributions to the study of language in MCI, this Research Topic groups together five papers that present the main advances in the detection of lexical deterioration in the early stages of dementia, particularly in MCI. Maruta and Pavão Martins have contributed a new approach to a longitudinal study to examine the predictive value of subjective language complaints (SLC) for cognitive decline in healthy, community-dwelling, older adults. They explored language complaints (SLC) and determined relationships between SLC and objective decline in verbal fluency tests at baseline and over time. They found significant differences in semantic fluency measures, between complaints and non-complaint groups, even though longitudinal slopes were not associated with an outcome of objective cognitive decline/dementia. Quaranta et al. proposed another longitudinal study that investigated the semantic relations in MCI by using a categorical verbal fluency test. It is well-known that semantic and conceptual knowledge is affected in dementia (Kemper and Altmann, 2009), however, the deterioration of these functions in MCI is at present underexplored. Although a semantic deficit seems to be present in MCI (Taler and Phillips, 2008), it is unclear if it affects the semantic representations or rather the process that gives access to them. The authors used a sophisticated analysis of semantic similarities and relatedness and found that the structure of semantic relations was weakened in MCI patients who converted to dementia. Lexical semantic impairments was also the topic of the paper by Taler et al.. In a cross-sectional design using several tasks to assess lexical access, retrieval, and the recognition of semantic information, the authors found that access to the phonological and orthographic lexicon was more affected than the semantic knowledge and retrieval in MCI. Lexical access in the early stages of dementia was also addressed by Campos-Magdaleno et al. in a longitudinal design, which studied the tip-of-the-tongue phenomenon. Results showed that deficits in phonological access successfully differentiated stable and worsened MCI patients from adults with subjective complaints. In light of the transmission deficit hypothesis (Burke et al., 1991) and in-line with previous cross-sectional studies, the authors explained lexical deterioration as a continuum that begins in normal aging with a deficit in phonological access and follows in MCI with a significant increment of that deficit and relative maintenance of semantic knowledge and access (Juncos-Rabadán et al., 2014).

Finally, Chen et al. proposed improving the evaluation of the semantic retrieval process in MCI with a novel multi-feature automated analysis of verbal fluency. Through an innovative machine-learning study, the authors incorporated time-based measures of a semantic fluency task, in addition to the traditional word count, to characterize semantic search strategies. Their promising results indicated that their computational model has high predictive validity in distinguishing MCI from cognitively unimpaired participants.

Research efforts in the study of language, not only at semantic and lexical level but also in terms of syntax and discourse, in the early stages of dementia, continue to find reliable and valid markers of diagnosis and progression. We believe that significant advances in this field in the future will come from further improvement of the diagnostic criteria and application of analytical procedures for big data.

## Author Contributions

All authors listed have made a substantial, direct and intellectual contribution to the work, and approved it for publication.

## Conflict of Interest

The authors declare that the research was conducted in the absence of any commercial or financial relationships that could be construed as a potential conflict of interest.
